# The longitudinal association of common susceptibility variants for type 2 diabetes and obesity with fasting glucose level and BMI

**DOI:** 10.1186/1471-2350-11-140

**Published:** 2010-10-08

**Authors:** Rebecca J Webster, Nicole M Warrington, John P Beilby, Timothy M Frayling, Lyle J Palmer

**Affiliations:** 1 Centre for Genetic Epidemiology and Biostatistics, University of Western Australia, Crawley, WA, Australia; 2 Genetics of Complex Traits, Peninsula Medical School, University of Exeter, Exeter, UK; 3 Pathology and Laboratory Medicine, University of Western Australia, Crawley, WA, Australia; 4 Molecular Genetics, PathWest Laboratory Medicine, Nedlands, WA, Australia

## Abstract

**Background:**

Variation in the effects of genetic variants on physiological traits over time or with age may alter the trajectories of these traits. However, few studies have investigated this possibility for variants associated with type 2 diabetes or obesity, and these show little consensus. We aimed to characterise the possible longitudinal associations of common diabetes-susceptibility variants in the *KCNJ11*, *PPARG*, *TCF7L2, IGF2BP2, CDKAL1, SLC30A8 *and *HHEX *gene loci, with fasting glucose level; and of an obesity-associated variant in the *FTO *gene, with body mass index (BMI).

**Methods:**

The study analysed data from the Busselton Health Study (*n *= 4,554). Cross-sectional association analyses included family data and used the total association test. Longitudinal association analyses of unrelated participant data (*n *= 2,864) used linear mixed-effects models.

**Results:**

In cross-sectional analyses, we observed associations of the T allele at the *IGF2BP2 *single nucleotide polymorphism (SNP) rs4402960 with raised fasting glucose (*p *= 0.045), and the A allele at the *FTO *SNP rs9939609 with raised BMI (*p *= 0.003). Longitudinal analyses showed no significant associations between SNPs and changes in fasting glucose or BMI in the same individuals, either over mean follow-up times of 18.7 and 21.8 years respectively, or with age during adulthood.

**Conclusions:**

There was no indication that the effects of common type 2 diabetes variants on fasting glucose varied with age during adulthood or over time.

## Background

Physiological traits are frequently dynamic, varying over time with changing or accumulating environmental and physiological factors. The influence of genotype on these traits may also vary over time through interaction with factors such as age, developmental stage or time-dependent environmental factors. Variation in the effects of genetic variants at different stages of life could significantly alter the trajectories of traits and potentially also the risks of certain diseases. Hence, studies that do not consider the possibility of longitudinal variation in genetic associations may lead to over-simplistic models of variant effects.

Type 2 diabetes (T2D) is a complex disease with numerous risk factors, including a growing number of known genetic susceptibility variants [1-4]. The risk of T2D also rises with increasing age [5]. Some of the intermediate traits of T2D, such as obesity and raised fasting plasma glucose, similarly have complex determinants and indeed both BMI and fasting plasma glucose also tend to increase with age [6,7]. These observations raise the question of whether time- or age-varying genetic effects exist that could influence such intermediate traits and thereby T2D risk.

To date, there have been few studies of the longitudinal effects of T2D- or obesity-susceptibility variants. Those that have examined the longitudinal effects of the *FTO *variant rs9939609 with BMI, give mixed results [8-13]. This SNP has been shown through cross-sectional studies to be robustly associated with raised BMI and, likely as a result of this effect, also linked to the risk of T2D [3,11,14-16]. Some longitudinal studies also provide evidence of an age-dependent relationship between rs9939609 and BMI whilst others do not, and some were performed in children and some in adults [8-13].

In the present study, we tested the cross-sectional and longitudinal associations of common genetic variants shown to be associated with the risk of T2D and/or raised BMI [1-4,14,15,17-20], with fasting glucose levels and BMI. The study utilised data from the Busselton Health Study (BHS), in which participants have completed an average of 3.9 phenotypic surveys each, over an average follow-up time of 21.2 years (95% CI 13.6-28.8 years). The variants selected were the potassium inwardly-rectifying channel J11 (*KCNJ11*) single nucleotide polymorphism (SNP) rs5219 (E23K variant); the peroxisome proliferator-activated receptor gamma (*PPARG*) SNP rs1801282 (Pro12Ala variant); the transcription factor 7-like 2 (*TCF7L2*) SNP rs7903146; the insulin-like growth factor 2 mRNA binding protein 2 (*IGF2BP2*) SNP rs4402960; the CDK5 regulatory subunit associated protein1-like 1 (*CDKAL1*) SNP rs10946398; the solute carrier family 30 (zinc transporter), member 8 (*SLC30A8*) SNP rs13266634; the hematopoietically expressed homeobox (*HHEX*) SNP rs1111875; and the fat mass and obesity associated (*FTO*) SNP rs9939609. These variants represent those with the strongest reported effects on type 2 diabetes risk and, for the *FTO *variant, BMI.

## Methods

### Participants

Participants were part of the BHS. The BHS includes a series of seven cross-sectional population health surveys of adult residents of the Shire of Busselton in Western Australia, conducted between 1966 and 1990, plus a follow-up study of all survivors of previous surveys, conducted in 1994/95 [21-23]. The population is predominantly European-Australian. The 1994/95 follow-up study involved 4,554 participants from 696 families, and included the collection of blood samples to be used for DNA extraction. Population descriptives for participants surveyed in the 1994/95 follow-up have been presented elsewhere [24] and are reproduced here in Table 1 with kind permission of Springer Science+Business Media. 2,864 unrelated individuals, each of whom had participated in at least one of the eight cross-sectional surveys while aged between 18 and 80 years, were selected from this cohort for longitudinal analysis. A subset of this unrelated sub-cohort, comprising only the 2,583 individuals that reported never having been diagnosed with diabetes and had no fasting glucose level measurements greater than or equal to 7 mmol/L, was also selected, for longitudinal analysis of the fasting glucose outcome variable only. Descriptives for individuals in the unrelated sub-cohort have been presented elsewhere [24] and are reproduced here in Table 2 with kind permission of Springer Science+Business Media. The mean number of survey attendances for these individuals was 3.9 (standard error of the mean 0.04), over a mean follow-up time of 21.2 years (95% confidence interval (CI) 13.6-28.8 years). Measurements of BMI, fasting glucose, triacylglycerol and high-density lipoprotein (HDL) levels were taken at 8, 7, 6 and 4 surveys respectively. The subset of unrelated participants had average follow-up times of 21.8, 18.7, 17.0 and 15.1 years for these measurements, respectively. All participants gave written informed consent. The Human Research Ethics Committee of the University of Western Australia approved all study protocols.

**Table 1:** Description of the BHS whole study population at the 1994/95 survey (*n *= 4,554).^a^

Variable (units)	Mean (SD)
Age (years)	50.6 (17.2)
BMI (kg/m^2^)	26.0 (4.2)
Fasting glucose (mmol/l)	5.0 (1.4)
Triacylglycerol (mmol/l)	1.3 (0.9)
LDL (mmol/l)	3.6 (1.0)
HDL (mmol/l)	1.4 (0.4)
Total cholesterol (mmol/l)	5.6 (1.1)
SBP (mmHg)	124.1 (17.9)
DBP (mmHg)	74.5 (10.2)
	
Male, % (*n*)	44.2 (2014)
Smoking (ever smoked), % (*n*)	17.7 (805)
Diabetes (ever had diabetes), % (*n*)	6.0 (271)
Obesity (BMI ≥ 30), % (*n*)	13.3 (726)
Coronary heart disease, % (*n*)	16.3 (744)
Metabolic syndrome (IDF definition), % (*n*)	19.1 (871)
Metabolic syndrome (NCEP definition), % (*n*)	15.9 (722)
Lipid-lowering medication, % (*n*)	2.5 (116)
Insulin injections, % (*n*)	0.7 (31)

IDF, International Diabetes Federation; NCEP, National Cholesterol Education Program

^a ^Originally published as Table 1 in Webster RJ, Warrington NM, Weedon MN, Hattersley AT, McCaskie PA, Beilby JP, Palmer LJ, Frayling TM: The association of common genetic variants in the APOA5, LPL and GCK genes with longitudinal changes in metabolic and cardiovascular traits. *Diabetologia *2009, **52**(1):106-114, ^© ^Springer-Verlag 2008, reproduced here with kind permission of Springer Science+Business Media.

**Table 2:** Description of BHS populations of participants aged 18-80 years, for variables with longitudinal data, at the time of first assessment.^a^

Variable (units)	Unrelated population (*n *= 2,864), mean (SD)	Unrelated male population (*n *= 1,234), mean (SD)	Unrelated female population (*n *= 1,630), mean (SD)
Age (years)	37.8 (11.6)	38.1 (11.6)	37.6 (11.6)
BMI (kg/m^2^)	24.3 (3.6)	24.9 (3.2)	23.8 (3.8)
Fasting glucose (mmol/l)	5.0 (1.1)	5.2 (1.4)	4.8 (0.8)
Triacylglycerol (mmol/l)	1.2 (0.8)	1.4 (1.0)	1.1 (0.6)
HDL (mmol/l)	1.4 (0.4)	1.3 (0.3)	1.6 (0.4)
Total cholesterol (mmol/l)	5.6 (1.2)	5.6 (1.2)	5.6 (1.2)

^a ^Originally published as Table 2 in Webster RJ, Warrington NM, Weedon MN, Hattersley AT, McCaskie PA, Beilby JP, Palmer LJ, Frayling TM: The association of common genetic variants in the APOA5, LPL and GCK genes with longitudinal changes in metabolic and cardiovascular traits. *Diabetologia *2009, **52**(1):106-114, ^© ^Springer-Verlag 2008, reproduced here with kind permission of Springer Science+Business Media.

### Clinical measurements

Clinical measurements were taken as described previously [21,22]. A self-administered questionnaire was used to determine participants' history of smoking, physician-diagnosed diabetes and use of lipid-lowering medication and insulin injections. Anthropomorphic measurements were taken according to standard clinical procedures. The presence of coronary heart disease was determined using the Rose questionnaire, electrocardiogram and self-reported history of physician-diagnosed angina or myocardial infarction.

### Laboratory measurements

Fasting glucose, insulin and lipid (HDL, low-density lipoprotein (LDL) and triacylglycerol) levels were measured from fasting venous blood samples as described previously [25]. The HOMA Calculator [26] was used to calculate homeostatic model assessment (HOMA) scores to be used as indicators of insulin resistance. These were HOMA2-%B (steady-state beta cell function) and HOMA2-%S (insulin sensitivity) [27,28].

### Genes and genotyping

The SNPs selected for this study were the *KCNJ11 *SNP rs5219; the *PPARG *SNP rs1801282; the *TCF7L2 *SNP rs7903146; the *IGF2BP2 *SNP rs4402960; the *CDKAL1 *SNP rs10946398; the *SLC30A8 *SNP rs13266634; the *HHEX *SNP rs1111875; and the *FTO *SNP rs9939609. These SNPs were genotyped for all 4,554 study participants using TaqMan probes designed and supplied by Applied Biosystems, Scoresby, VIC Australia. All primer sequences and experimental conditions are available from the authors on request. Genotyping was performed by the PathWest Molecular Genetics Service, Nedlands, WA, Australia.

### Statistical analyses

The statistical analysis approach and methods used for this study are as described in detail elsewhere [24]. Tests of Hardy-Weinberg equilibrium were conducted using an exact test implemented in the statistical analysis package, R [29].

Cross-sectional association analyses were performed using 1994/95 survey data from the whole cohort of 4,554 participants. The continuous outcome variables tested were HDL, LDL, triacylglycerol, systolic blood pressure (SBP), diastolic blood pressure (DBP), fasting glucose and fasting insulin levels, HOMA2-%B, HOMA2-%S, and BMI. With the exception of LDL and DBP, all were natural log-transformed prior to analysis. The binary outcome variables tested were 'history of diabetes', a variable indicating whether a participant had reported ever been diagnosed with diabetes at any survey, and 'obesity', a variable indicating whether a participant was obese (BMI ≥ 30) at the 1994/95 survey. Selection of covariates for each outcome involved the use of generalised linear models to model the effects of a set of multiple relevant phenotypes on the outcome in the unrelated sub-cohort, using the SimHap v1.0.0 program http://www.genepi.org.au/simhap.html, and a process of stepwise elimination to remove those not associated with the outcome. Models derived using this procedure were called full covariate models. Simple covariate models including only sex and age were also used for analyses. Cross-sectional association analyses of continuous outcomes employed the total association test as implemented in the QTDT v2.6.0 program [30]. This test models the effects of covariates on continuous outcomes within families. Between-individual variance was modelled by environmental, polygenic and additive major-gene-effect variance components. Cross-sectional analysis of the binary obesity outcome employed the transmission disequilibrium test (TDT) as implemented in the QTDT v2.6.0 program. As there were too few participants with a history of diabetes in the whole cohort to enable a TDT of family data to be performed for all SNPs, cross-sectional analyses of the history of diabetes outcome were performed on the sub-cohort of unrelated participants aged 18-80 years, using the SimHap v1.0.0 program to fit generalised linear models.

Longitudinal association analyses were performed using data from the sub-cohort of unrelated participants aged 18-80 years and, for the case of the fasting glucose outcome variable only, on the non-diabetic subset. The outcome variables tested were fasting glucose, HDL and triacylglycerol levels, and BMI, which were natural log-transformed prior to analysis. The age, time and BMI covariates were mean-centred. Longitudinal association analyses were conducted using linear mixed-effects models [31] with time × SNP and age × SNP interaction terms, using the SimHap program. Covariates were selected through a process of stepwise elimination as described above. Genotypes were coded into three classes (major allele homozygote = 0, heterozygote = 1 or minor allele homozygote = 2) and analysed under a co-dominant genetic model. The results were used to identify significant associations and investigate the most appropriate genetic model for each case. The results of the co-dominant model analyses are presented due to the difficulty in assigning a more appropriate model in the majority of cases.

### Statistical power

All power calculations were performed using the Quanto v1.2.3 software [32]. For all SNPs, analyses of BMI and fasting glucose had at least 80% power at an alpha level of *p *= 0.05 to detect a difference in beta coefficient of 0.010 and 0.013 (log-transformed units) respectively, for the case of longitudinal analyses of the unrelated sub-cohort, and 0.014 and 0.011 (log-transformed units) respectively, for the case of cross-sectional analyses of the whole cohort, under additive genetic models.

## Results

### Population characteristics

Genotypic and allelic frequencies for all eight SNPs in the BHS populations are given in Additional file 1: Electronic Supplementary Material (ESM) Table 1. No significant deviation of allele frequencies from Hardy-Weinberg equilibrium was observed in either the whole study cohort or the unrelated sub-cohort for any of the SNPs. Call rates for each SNP, validated through sequencing, are also given in Additional file 1: ESM Table 1. Call rates exceeded 99% for all SNPs.

### Cross-sectional association of SNPs with metabolic and cardiovascular traits

The results of cross-sectional total association analyses of the whole cohort (*n *= 4,554) at the 1994/95 survey for the fasting glucose and BMI outcomes are given in Table 3. In analyses using full covariate models, we observed that the T T2D-risk allele at the *IGF2BP2 *SNP rs4402960 was associated with raised fasting glucose level (*p *= 0.045), and that the A obesity-risk allele at the *FTO *SNP rs9939609 was associated with raised BMI (*p *= 0.003). Similar results were obtained using the simple covariate model. In multivariate cross-sectional analyses of traits in the sub-cohort of unrelated individuals at the 1994/95 survey, we observed that, under an additive genetic model, rs4402960 explained 0.1% of variation in fasting glucose level in unrelated non-diabetic participants (*n *= 2,583). There was no significant association between BMI and rs9939609 in the unrelated sub-cohort at the 1994/95 survey (*n *= 2,864), and the percentage of variance in BMI explained by rs9939609 in this sub-cohort was estimated to be 0%.

**Table 3:** Results of cross-sectional association analyses of fasting glucose and BMI.^a^

			Full covariate model^b^		Simple covariate model^c^	
				
Gene	SNP	Risk allele (frequency)	Per risk allele effect size	*p*	Per risk allele effect size	*p*
**Fasting glucose**						
*KCNJ11*	rs5219	T (0.363)	0.001	0.70	-0.001	0.75
*PPARG*	rs1801282	C (0.883)	0.003	0.56	0.011	0.05
*TCF7L2*	rs7903146	T (0.303)	0.003	0.44	0.006	0.12
*IGF2BP2*	rs4402960	T (0.304)	0.007	0.045*	0.011	0.01*
*CDKAL1*	rs10946398	C (0.303)	0.006	0.08	0.003	0.42
*SLC30A8*	rs13266634	C (0.699)	0.007	0.05	0.005	0.18
*HHEX*	rs1111875	G (0.584)	-0.002	0.51	-0.003	0.40
**BMI**						
*FTO*	rs9939609	A (0.409)	0.009	0.003*	0.013	0.0003*

^a ^Analyses were conducted on data from the whole study population (*n *= 4,554) at the 1994/95 survey.

^b ^Phenotype adjustments, full covariate model:

Fasting glucose: sex, age, age squared, BMI, fasting insulin, SBP, history of diabetes

BMI: sex, age, age squared, fasting insulin, HDL, SBP, DBP

^c ^Phenotype adjustments for all outcomes, simple covariate model: sex, age

* *p *< 0.05

The results of cross-sectional total association analyses performed with additional metabolic and cardiovascular outcomes are given in Additional file 1: ESM Table 2. In analyses using full covariate models, we observed that the C T2D-risk allele at the *CDKAL1 *SNP rs10946398 was associated with reduced HOMA2-%B (*p *= 0.01); that the major C T2D-risk allele at the *SLC30A8 *SNP rs13266634 was associated with reduced HOMA2-%B (*p *= 0.03); that the major C T2D-risk allele at the *PPARG *SNP rs1801282 was associated with reduced HOMA2-%S (*p *= 0.01) and raised fasting insulin level (*p *= 0.01); that the major G T2D-risk allele at the *HHEX *SNP rs1111875 was associated with raised HOMA2-%S (*p *= 0.01) and reduced fasting insulin level (*p *= 0.01); that the T T2D-risk allele at the *KCNJ11 *SNP rs5219 was associated with reduced BMI (*p *= 0.001); that the T T2D-risk allele the *TCF7L2 *SNP at rs7903146 was associated with raised triacylglycerol (*p *= 0.01) and LDL levels (*p *= 0.01); and that the T T2D-risk allele at the *IGF2BP2 *SNP rs4402960 was associated with raised triacylglycerol level (*p *= 0.001). Similar results were obtained using the simple covariate model (data not shown).

The results of cross-sectional association analyses of the binary outcomes history of diabetes, and obesity are given in Additional file 1: ESM Table 3. The C T2D-risk allele at the *PPARG *SNP rs1801282, the T T2D-risk allele at the *TCF7L2 *SNP rs7903146, and the T T2D-risk allele at the *IGF2BP2 *SNP rs4402960 were significantly associated with T2D (*p *= 0.0496, *p *= 0.039, *p *= 0.022, respectively), with odds ratios of 1.46 (95% CI 1.00-2.12), 1.27 (95% CI 1.01-1.60) and 1.30 (95% CI 1.04-1.63) per risk allele, respectively. There was no significant association of the *KCNJ11 *SNP rs5219, the *CDKAL1 *SNP rs10946398, the *SLC30A8 *SNP rs13266634, the *HHEX *SNP rs1111875, or the *FTO *SNP rs9939609 with T2D. Analysis of the obesity outcome showed that the *FTO *SNP rs9939609 was not significantly associated with obesity (*p *= 0.457). Similar results were obtained using the simple covariate model (data not shown).

### Longitudinal association of SNPs with metabolic and cardiovascular traits

**Figure 1:**
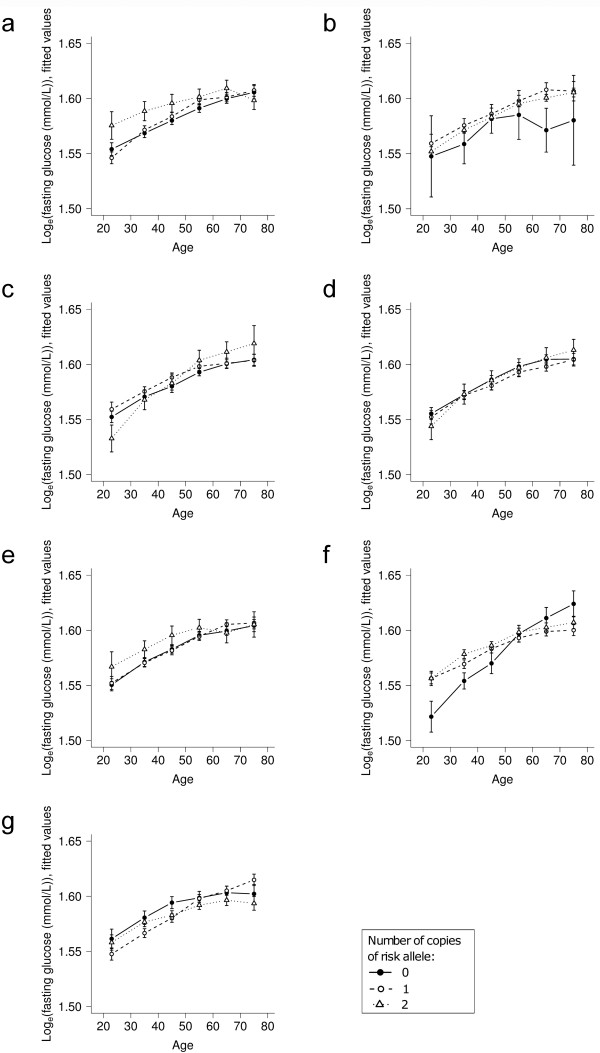
**Variation with age of adjusted, natural log-transformed fasting glucose by genotype**. Data is from unrelated, non-diabetic participants aged 18-80 years (*n *= 2,583), for seven SNPs: (a) *KCNJ11 *SNP rs5219, (b) *PPARG *SNP rs1801282, (c) *TCF7L2 *SNP rs7903146, (d) *IGF2BP2 *SNP rs4402960, (e) *CDKAL1 *SNP rs10946398, (f) *SLC30A8 *SNP rs13266634, (g) *HHEX *SNP rs1111875. Values were fitted using co-dominant genetic models.

**Figure 2:**
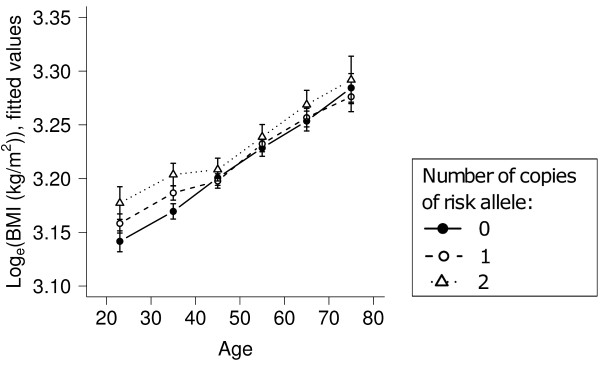
**Variation with age of adjusted, natural log-transformed BMI by rs9939609 genotype**. Data is from unrelated participants aged 18-80 years (*n *= 2,864). Values were fitted using a co-dominant genetic model.

The results of longitudinal association analyses for the fasting glucose level and BMI outcomes are given in Table 4. For each of these analyses, fitted values, calculated according to the co-dominant genetic models used, are plotted by genotype group against age in Figures 1 and 2.

**Table 4:** Results of longitudinal association analyses of fasting glucose and BMI.^a^

			Age × SNP interaction		Time × SNP interaction	
				
Gene	SNP (Risk allele, frequency)	Factor	Beta (95% CI)	*p*	Beta (95% CI)	*p*
**Fasting glucose^b ^(log_e_[mmol/l])**						
*KCNJ11*	rs5219	CC	Baseline			
	(T, 0.355)	CT	7.0 × 10^-5 ^(-4.6 × 10^-4^, 6.0 × 10^-4^)	0.79	-5.3 × 10^-4 ^(-1.3 × 10^-3^, 2.6 × 10^-4^)	0.19
		TT	-4.3 × 10^-4 ^(-1.2 × 10^-3^, 3.3 × 10^-4^)	0.27	-6.8 × 10^-4 ^(-1.8 × 10^-3^, 4.8 × 10^-4^)	0.25
*PPARG*	rs1801282	CC	Baseline			
	(C, 0.881)	CG	8.0 × 10^-5 ^(-5.2 × 10^-4^, 6.8 × 10^-4^)	0.79	-3.6 × 10^-4 ^(-1.3 × 10^-3^, 5.4 × 10^-4^)	0.43
		GG	-8.8 × 10^-4 ^(-3.0 × 10^-3^, 1.2 × 10^-3^)	0.41	3.7 × 10^-4 ^(-2.8 × 10^-3^, 3.6 × 10^-3^)	0.82
*TCF7L2*	rs7903146	CC	Baseline			
	(T, 0.300)	CT	-1.2 × 10^-4 ^(-6.3 × 10^-4^, 4.0 × 10^-4^)	0.65	-3.2 × 10^-4 ^(-1.1 × 10^-3^, 4.5 × 10^-4^)	0.41
		TT	7.3 × 10^-4 ^(-1.6 × 10^-4^, 1.6 × 10^-3^)	0.11	5.6 × 10^-4 ^(-8.0 × 10^-4^, 1.9 × 10^-3^)	0.42
*IGF2BP2*	rs4402960	GG	Baseline			
	(T, 0.314)	GT	-3.8 × 10^-5 ^(-5.5 × 10^-4^, 4.8 × 10^-4^)	0.89	6.3 × 10^-4 ^(-1.5 × 10^-4^, 1.4 × 10^-3^)	0.12
		TT	4.7 × 10^-4 ^(-4.1 × 10^-4^, 1.4 × 10^-3^)	0.30	-4.0 × 10^-4 ^(-1.7 × 10^-3^, 9.1 × 10^-4^)	0.55
*CDKAL1*	rs10946398	AA	Baseline			
	(C, 0.308)	AC	4.9 × 10^-5 ^(-4.7 × 10^-4^, 5.7 × 10^-4^)	0.85	2.1 × 10^-4 ^(-5.7 × 10^-4^, 9.9 × 10^-4^)	0.60
		CC	-3.3 × 10^-4 ^(-1.2 × 10^-3^, 5.0 × 10^-4^)	0.43	-3.6 × 10^-4 ^(-1.6 × 10^-3^, 9.1 × 10^-4^)	0.58
*SLC30A8*	rs13266634	CC	Baseline			
	(C, 0.696)	CT	2.4 × 10^-5 ^(-4.9 × 10^-4^, 5.4 × 10^-4^)	0.93	1.4 × 10^-4 ^(-6.4 × 10^-4^, 9.1 × 10^-4^)	0.73
		TT	8.0 × 10^-4 ^(-5.2 × 10^-5^, 1.7 × 10^-3^)	0.07	-1.2 × 10^-4 ^(-1.4 × 10^-3^, 1.2 × 10^-3^)	0.86
*HHEX*	rs1111875	GG	Baseline			
	(G, 0.578)	GA	6.1 × 10^-4 ^(6.3 × 10^-5^, 1.2 × 10^-3^)	0.03*	-2.3 × 10^-4 ^(-1.0 × 10^-3^, 6.0 × 10^-4^)	0.59
		AA	-2.3 × 10^-4 ^(-9.3 × 10^-4^, 4.8 × 10^-4^)	0.53	2.4 × 10^-4 ^(-8.2 × 10^-4^, 1.3 × 10^-3^)	0.65
**BMI^b ^(log_e_[kg/m^2^])**						
*FTO*	rs9939609	TT	Baseline			
	(A, 0.402)	TA	2.6 × 10^-4 ^(-4.7 × 10^-4^, 9.9 × 10^-4^)	0.49	2.1 × 10^-5 ^(-7.7 × 10^-4^, 8.1 × 10^-4^)	0.96
		AA	-2.0 × 10^-5 ^(-1.0 × 10^-3^, 9.9 × 10^-4^)	0.97	-2.8 × 10^-4 ^(-1.4 × 10^-3^, 8.1 × 10^-4^)	0.61

^a ^BMI outcome analyses were conducted on data from unrelated participants aged 18-80 years (*n *= 2,864). Fasting glucose outcome analyses were conducted on data from the subset of non-diabetic participants (*n *= 2,583). Results are for age × SNP and time × SNP interaction terms with co-dominant genetic models.

^b ^Phenotype adjustments:

Fasting glucose: sex, age, BMI, age at first survey, time of survey

BMI: sex, age, age × sex, smoking status, age at first survey, time of survey

*p < 0.05

For longitudinal analyses of the fasting glucose level outcome, the SNP simple effects (non-interaction) results showed no significant associations of any of the seven SNPs with fasting glucose in the unrelated sub-cohort (data not shown). The SNP interaction results showed no strong evidence of effect sizes varying with age (from 18-80 years) or time (from 1975-1994/5). Analysis of rs1111875 with fasting glucose under a co-dominant model showed a significant association of the age × rs1111875 (heterozygote vs major homozygote) interaction term with fasting glucose level (Table 4; *p *= 0.03), suggesting that one copy of the A non-risk allele at rs1111875 increases fasting glucose level with increasing age. However, under an additive genetic model, deemed to be the most likely genetic model for this particular association based on the co-dominant model results, the age × rs1111875 interaction term was not significantly associated with fasting glucose level, indicating that there is no significant overall linear trend in the effect size of rs1111875 genotype on fasting glucose level with age.

In longitudinal analyses of BMI, rs9939609 simple effects were found to be significant, with the rs9939609 minor homozygote vs major homozygote term being significantly associated with raised BMI under a co-dominant model (*p *= 0.003), and the rs9939609 term being significantly associated with raised BMI under the best-fitting recessive model (*p *= 0.004). This indicates that the A allele at rs9939609 is significantly associated with raised BMI in the unrelated sub-cohort, when data from all surveys are considered. However, there was no evidence for longitudinal variation in the effect size of rs9939609 genotype on BMI, either over time or with age from 18-80 years.

Longitudinal association analysis results for lipid level and BMI outcomes with all SNPs are given in Additional file 1: ESM Table 4. We observed some evidence for increasing effect sizes of the T risk alleles at rs7903146 and rs4402960 on triacylglycerol level with time and age respectively, and for a decreasing effect size of the C risk allele at rs13266634 on triacylglycerol level with age. We also observed some evidence that the effect sizes of the risk C alleles at rs1801282 and rs13266634 on HDL varied with both age and time, a decreasing effect with age and increasing effect with time for the case of rs1801282, and an increasing effect with age and decreasing effect with time for the case of rs13266634. There was also some evidence that the effect size of the G risk allele at rs1111875 on BMI decreased with age and increased with time.

## Discussion

While the number of genetic variants confirmed to be associated with T2D is growing, the relationships between these variants and T2D intermediate phenotypes are yet to be fully characterised, particularly longitudinally. In this study, we investigated the cross-sectional and longitudinal associations of seven T2D-susceptibility variants in the *KCNJ11*, *PPARG*, *TCF7L2, IGF2BP2, CDKAL1, SLC30A8 *and *HHEX *genes with fasting glucose level, and of another in the *FTO *gene with BMI, in a large, population-based European-Australian cohort.

Our cross-sectional analyses showed that, of the seven SNPs analysed with the fasting glucose outcome, only the *IGF2BP2 *SNP rs4402960 showed a significant result, the T allele at this SNP being nominally associated with raised fasting glucose (*p *= 0.045). Though some previous studies have reported significant associations of rs4402960, rs7903146 and rs13266634 with raised fasting glucose [33-36], others find no significant associations between these three SNPs and fasting glucose in their populations [33,34,37-40]. Studies of the SNPs rs5219, rs1801282, rs10946398 and rs1111875 have also shown no significant associations with fasting glucose [17,33,34,38,40-44]. Rather, the majority of evidence from previous studies suggests that these seven SNPs alter T2D susceptibility through effects on insulin sensitivity (rs1801282 [45]) or pancreatic beta cell function (rs5219, rs7903146, rs10946398, rs4402960, rs13266634 and rs1111875 [34,36,38,46-52]). Indeed, we did observe associations of the risk allele at the *PPARG *SNP rs1801282 with lower HOMA2-%S (*p *= 0.01), and of the risk alleles at the *CDKAL1 *SNP rs10946398 and the *SLC30A8 *SNP rs13266634 with lower HOMA2-%B (*p *= 0.01 and *p *= 0.03, respectively). Fasting glucose level may reflect abnormalities in insulin sensitivity and beta cell function, and is a useful clinical indicator of diabetes. However, it is dependent on a number of other factors, e.g. the site of insulin insensitivity [53], which may have influenced the results here. It may also be that effects on fasting glucose are more subtle than may be detected with the available power. We also did not observe significant associations of the SNPs rs5219, rs10946398, rs1111875 or rs9939609 with T2D, likely due in part to the small number of diabetic participants in our cohort.

Associations may also be more difficult to detect if SNP effects vary with factors such as other gene variants, lifestyle factors and age. If a SNP has an age-varying effect on a particular trait, such as an effect to raise fasting glucose level as of middle age for example, then cross-sectional analysis of participants of all ages may miss the association. On the other hand, longitudinal analysis may still identify the relationship, by examining the association of the combination of SNP allele and age with phenotype in aging individuals, through a SNP × age interaction term. However, in our longitudinal analyses of SNPs with fasting glucose level, we found no evidence of changes in associations either with age or over time, indicating that these SNPs are not involved in the age-related increase in fasting glucose level, and that their effects are not influenced by any environmental factors that have changed over the ~20 year time period examined. The curves of Figure 1 depict these results, generally showing no significant differences in the rates at which fasting glucose level increases with age for the three genotype groups of each SNP, although it is important to note again that we did not see strong evidence for cross-sectional differences.

Our investigation of the *FTO *SNP rs9939609 showed no significant association with obesity. However, we did confirm that the A allele at rs9939609 was significantly associated with raised BMI, both in cross-sectional analyses of the whole adult study population (*p *= 0.003), and in analyses of the unrelated sub-cohort performed across all surveys (*p *= 0.004). The association between *FTO *variants and BMI is not in doubt, and has been described both in childhood and old age [11,14-16,54]. For the rs9939609 SNP, an effect size of about 0.4 kg/m^2 ^per susceptibility allele has been reported [14]. But does this effect size alter with age? We found no significant evidence of this in our analysis of the rs9939609 SNP in the unrelated study population. Several other studies of the longitudinal effects of *FTO *on BMI have been conducted. Some of these have reported different effects at different ages, but these findings were often limited to one sex or another subgroup after a primary analysis, and chance may have influenced these results [8,11-13]. The evidence that *FTO *has different effects on BMI at different ages or at different times is therefore weak and further studies are needed.

Differences between the findings of this and other studies may have arisen from numerous sources. Most likely chance has played a strong role. Moreover, this study is underpowered to detect small to moderate genetic effects. Hence, the failure to observe significant associations must be interpreted in this light. Differences in factors that may interact with genotype but were not controlled for, e.g. lifestyle factors such as diet and physical activity, may also have influenced the results, as may have differences in the ethnic compositions of the cohorts studied. The risk allele frequencies of all eight SNPs in the BHS cohort studied here (Additional file 1: ESM Table 1) are comparable to those in European populations, as reported in the dbSNP database http://www.ncbi.nlm.nih.gov/projects/SNP/.

It is clear that further studies are required to assess the validity of our longitudinal findings. These would ideally address the issues of analytic power and additional covariates, and test the generalisability of our results to populations from diverse ethnic backgrounds. In addition, though the average follow-up times of 18.7 years for fasting glucose level and 21.8 years for BMI enable an extended longitudinal analysis to be conducted, they cannot provide as complete a picture of age-related changes as would come from a study that followed participants through from youth to old age. However, to our knowledge, few longer running longitudinal surveys of the same individuals are able to address these longitudinal questions, and the BHS provides phenotypic data that is particularly comprehensive. The understanding of the function of the T2D-susceptibility SNPs studied here would also benefit from longitudinal analysis of additional metabolic traits, such as glucose tolerance test 30 minute and 2 hour glucose levels, which would provide further information on their relationship to impaired glucose tolerance.

## Conclusions

In conclusion, the results of this study showed no indication that the effects of seven common T2D susceptibility variants on fasting glucose level or the effect of the *FTO *SNP rs9939609 on BMI varied with age during adulthood or over time. However, the results should be interpreted with caution due to power considerations. The potential consequences of longitudinal associations on phenotypic traits and their treatment across the lifespan highlight the need for replication and further characterisation studies.

## List of abbreviations

BHS: Busselton Health Study; BMI: body mass index; CI: confidence interval; DBP: diastolic blood pressure; HDL: high-density lipoprotein; HOMA: homeostatic model assessment; LDL: low-density lipoprotein; SBP: systolic blood pressure; SNP: single nucleotide polymorphism; T2D: type 2 diabetes.

## Competing interests

The authors declare that they have no competing interests.

## Authors' contributions

RW carried out the statistical analyses and drafted the manuscript. NW contributed statistical advice for the cross-sectional and longitudinal analyses. JB was involved in data collection and laboratory analysis. TF participated in the design and coordination of the study and helped to draft the manuscript. LP participated in the design and coordination of the study. All authors read and approved the final manuscript.

## Pre-publication history

The pre-publication history for this paper can be accessed here:

http://www.biomedcentral.com/1471-2350/11/140/prepub

## Supplementary Material

Additional file 1 **Word document (376 k) containing Electronic Supplementary Material (ESM) Tables 1, 2, 3 and 4**. Click here for file
