# Supplementary material for: The longitudinal association of common susceptibility variants for type 2 diabetes and obesity with fasting glucose level and BMI
Source: BMC Med Genet. 2010 Oct 8;11:140. doi: 10.1186/1471-2350-11-140 (PMC2958899; doi:10.1186/1471-2350-11-140)
Supplement: Additional file 1 — Word document (376 k) containing Electronic Supplementary Material (ESM) Tables 1, 2, 3 and 4. [file 1471-2350-11-140-S1.DOC]

**ESM Table 1 – Genotypic and allelic frequencies in BHS populations.**

| Gene | SNP | Risk allele (frequency) | Homozygous  wild-type,  *n* (%; genotype) | Heterozygote,  *n* (%; genotype) | Homozygous mutant,  *n* (%; genotype) | Hardy-Weinberg equilibrium *p* | SNP  call rate |
| --- | --- | --- | --- | --- | --- | --- | --- |
| **Whole study population (*n*=4,554)** |  |  |  |  |  |  |  |
| *KCNJ11* | rs5219 | T (0.363) | 1853 (40.8%; CC) | 2076 (45.8%; CT) | 607 (13.4%; TT) | 0.500 | 99.9% |
| *PPARG* | rs1801282 | C(0.883) | 3513 (77.8%; CC) | 947 (21.0%; CG) | 57 (1.3%; GG) | 0.474 | 99.5% |
| *TCF7L2* | rs7903146 | T (0.303) | 2224 (49.1%; CC) | 1873 (41.3%; CT) | 436 (9.6%; TT) | 0.149 | 99.8% |
| *IGF2BP2* | rs4402960 | T (0.304) | 2202 (48.7%; GG) | 1893 (41.8%; GT) | 430 (9.5%; TT) | 0.440 | 99.7% |
| *CDKAL1* | rs10946398 | C (0.303) | 2210 (48.8%; AA) | 1889 (41.7%; AC) | 426 (9.4%; CC) | 0.439 | 99.7% |
| *SLC30A8* | rs13266634 | C (0.699) | 2210 (48.8%; CC) | 1905 (42.1%; CT) | 411 (9.1%; TT) | 1 | 99.6% |
| *HHEX* | rs1111875 | G (0.584) | 1568 (34.6%; GG) | 2155 (47.6%; GA) | 806 (17.8%; AA) | 0.169 | 99.7% |
| *FTO* | rs9939609 | A (0.409) | 1559 (34.7%; TT) | 2202 (48.9%; TA) | 738 (16.4%; AA) | 0.405 | 99.3% |
| **Unrelated,**  **18–80 year old population (*n*=2,864)** |  |  |  |  |  |  |  |
| *KCNJ11* | rs5219 | T (0.355) | 1194 (41.9%; CC) | 1293 (45.3%; CT) | 365 (12.8%; TT) | 0.624 | 99.9% |
| *PPARG* | rs1801282 | C (0.881) | 2202 (77.6%; CC) | 594 (20.9%; CG) | 40 (1.4%; GG) | 1 | 99.6% |
| *TCF7L2* | rs7903146 | T (0.300) | 1406 (49.3%; CC) | 1180 (41.3%; CT) | 265 (9.3%; TT) | 0.422 | 99.9% |
| *IGF2BP2* | rs4402960 | T (0.314) | 1346 (47.3%; GG) | 1217 (42.7%; GT) | 285 (10.0%; TT) | 0.664 | 99.8% |
| *CDKAL1* | rs10946398 | C (0.308) | 1369 (48.1%; AA) | 1200 (42.2%; AC) | 275 (9.7%; CC) | 0.629 | 99.7% |
| *SLC30A8* | rs13266634 | C (0.696) | 1375 (48.2%; CC) | 1215 (42.6%; CT) | 260 (9.1%; TT) | 0.691 | 99.8% |
| *HHEX* | rs1111875 | G (0.578) | 969 (34.0%; GG) | 1354 (47.5%; GA) | 525 (18.4%; AA) | 0.156 | 99.8% |
| *FTO* | rs9939609 | A (0.402) | 1014 (35.8%; TT) | 1358 (48.0%; TA) | 457 (16.1%; AA) | 0.969 | 99.5% |

**ESM Table 2 – Results of cross-sectional association analyses of continuous metabolic and cardiovascular phenotypes.^a^**

| Phenotype^b^ | Gene | SNP | Risk allele  (frequency) | Per risk allele effect size | *p* |
| --- | --- | --- | --- | --- | --- |
| HOMA2-%B | *KCNJ11* | rs5219 | T (0.363) | 0.001 | 0.69 |
|  | *PPARG* | rs1801282 | C (0.883) | 0.008 | 0.15 |
|  | *TCF7L2* | rs7903146 | T (0.303) | -0.005 | 0.20 |
|  | *IGF2BP2* | rs4402960 | T (0.304) | -0.007 | 0.43 |
|  | *CDKAL1* | rs10946398 | C (0.303) | -0.023 | 0.01^*^ |
|  | *SLC30A8* | rs13266634 | C (0.699) | -0.020 | 0.03^*^ |
|  | *HHEX* | rs1111875 | G (0.584) | -0.013 | 0.11 |
|  | *FTO* | rs9939609 | A (0.409) | -0.008 | 0.35 |
| HOMA2-%S | *KCNJ11* | rs5219 | T (0.363) | -0.018 | 0.11 |
|  | *PPARG* | rs1801282 | C (0.883) | -0.042 | 0.01^*^ |
|  | *TCF7L2* | rs7903146 | T (0.303) | 0.008 | 0.49 |
|  | *IGF2BP2* | rs4402960 | T (0.304) | -0.006 | 0.63 |
|  | *CDKAL1* | rs10946398 | C (0.303) | 0.012 | 0.31 |
|  | *SLC30A8* | rs13266634 | C (0.699) | 0.004 | 0.75 |
|  | *HHEX* | rs1111875 | G (0.584) | 0.030 | 0.01^*^ |
|  | *FTO* | rs9939609 | A (0.409) | 0.000 | 0.96 |
| Fasting insulin | *KCNJ11* | rs5219 | T (0.363) | 0.015 | 0.16 |
|  | *PPARG* | rs1801282 | C (0.883) | 0.041 | 0.01^*^ |
|  | *TCF7L2* | rs7903146 | T (0.303) | -0.012 | 0.30 |
|  | *IGF2BP2* | rs4402960 | T (0.304) | -0.005 | 0.66 |
|  | *CDKAL1* | rs10946398 | C (0.303) | -0.016 | 0.17 |
|  | *SLC30A8* | rs13266634 | C (0.699) | -0.008 | 0.47 |
|  | *HHEX* | rs1111875 | G (0.584) | -0.027 | 0.01^*^ |
|  | *FTO* | rs9939609 | A (0.409) | -0.004 | 0.72 |
| BMI | *KCNJ11* | rs5219 | T (0.363) | -0.010 | 0.001^*^ |
|  | *PPARG* | rs1801282 | C (0.883) | -0.007 | 0.15 |
|  | *TCF7L2* | rs7903146 | T (0.303) | -0.002 | 0.57 |
|  | *IGF2BP2* | rs4402960 | T (0.304) | 0.003 | 0.37 |
|  | *CDKAL1* | rs10946398 | C (0.303) | -0.002 | 0.46 |
|  | *SLC30A8* | rs13266634 | C (0.699) | -0.001 | 0.76 |
|  | *HHEX* | rs1111875 | G (0.584) | -0.004 | 0.20 |
|  | *FTO* | rs9939609 | A (0.409) | 0.009 | 0.003^*^ |
| Triacylglycerol | *KCNJ11* | rs5219 | T (0.363) | -0.004 | 0.72 |
|  | *PPARG* | rs1801282 | C (0.883) | -0.010 | 0.50 |
|  | *TCF7L2* | rs7903146 | T (0.303) | 0.027 | 0.01* |
|  | *IGF2BP2* | rs4402960 | T (0.304) | 0.034 | 0.001^*^ |
|  | *CDKAL1* | rs10946398 | C (0.303) | -0.007 | 0.51 |
|  | *SLC30A8* | rs13266634 | C (0.699) | 0.007 | 0.53 |
|  | *HHEX* | rs1111875 | G (0.584) | 0.006 | 0.55 |
|  | *FTO* | rs9939609 | A (0.409) | 0.001 | 0.90 |
| HDL | *KCNJ11* | rs5219 | T (0.363) | 0.002 | 0.73 |
|  | *PPARG* | rs1801282 | C (0.883) | -0.005 | 0.54 |
|  | *TCF7L2* | rs7903146 | T (0.303) | 0.006 | 0.27 |
|  | *IGF2BP2* | rs4402960 | T (0.304) | 0.004 | 0.42 |
|  | *CDKAL1* | rs10946398 | C (0.303) | -0.002 | 0.67 |
|  | *SLC30A8* | rs13266634 | C (0.699) | 0.005 | 0.56 |
|  | *HHEX* | rs1111875 | G (0.584) | -0.004 | 0.46 |
|  | *FTO* | rs9939609 | A (0.409) | 0.007 | 0.17 |
| LDL | *KCNJ11* | rs5219 | T (0.363) | 0.021 | 0.32 |
|  | *PPARG* | rs1801282 | C (0.883) | 0.017 | 0.59 |
|  | *TCF7L2* | rs7903146 | T (0.303) | 0.055 | 0.01^*^ |
|  | *IGF2BP2* | rs4402960 | T (0.304) | 0.008 | 0.73 |
|  | *CDKAL1* | rs10946398 | C (0.303) | 0.025 | 0.27 |
|  | *SLC30A8* | rs13266634 | C (0.699) | 0.026 | 0.22 |
|  | *HHEX* | rs1111875 | G (0.584) | -0.004 | 0.85 |
|  | *FTO* | rs9939609 | A (0.409) | 0.030 | 0.15 |
| SBP | *KCNJ11* | rs5219 | T (0.363) | 0.001 | 0.49 |
|  | *PPARG* | rs1801282 | C (0.883) | 0.001 | 0.65 |
|  | *TCF7L2* | rs7903146 | T (0.303) | -0.001 | 0.61 |
|  | *IGF2BP2* | rs4402960 | T (0.304) | 0.003 | 0.25 |
|  | *CDKAL1* | rs10946398 | C (0.303) | -0.001 | 0.77 |
|  | *SLC30A8* | rs13266634 | C (0.699) | -0.002 | 0.44 |
|  | *HHEX* | rs1111875 | G (0.584) | 0.001 | 0.60 |
|  | *FTO* | rs9939609 | A (0.409) | -0.000 | 0.86 |
| DBP | *KCNJ11* | rs5219 | T (0.363) | -0.129 | 0.45 |
|  | *PPARG* | rs1801282 | C (0.883) | 0.118 | 0.67 |
|  | *TCF7L2* | rs7903146 | T (0.303) | -0.056 | 0.76 |
|  | *IGF2BP2* | rs4402960 | T (0.304) | 0.090 | 0.63 |
|  | *CDKAL1* | rs10946398 | C (0.303) | 0.176 | 0.35 |
|  | *SLC30A8* | rs13266634 | C (0.699) | 0.089 | 0.63 |
|  | *HHEX* | rs1111875 | G (0.584) | 0.046 | 0.81 |
|  | *FTO* | rs9939609 | A (0.409) | 0.090 | 0.60 |

^a^ Analyses were conducted on data from the whole study population (*n*=4,554) at the 1994/95 survey.

^b^ Phenotype adjustments, full covariate model:

HOMA2-%B: sex, age, age squared, age cubed, BMI, WHR, HDL, triacylglycerol, history of diabetes

HOMA2-%S: sex, age, age squared, age cubed, BMI, BMI squared, BMI cubed, WHR, HDL, LDL, triacylglycerol, SBP, history of diabetes

Fasting insulin: sex, age, age squared, age cubed, BMI, BMI squared, BMI cubed, WHR, fasting glucose, HDL, LDL, triacylglycerol, SBP, history of diabetes

BMI: sex, age, age squared, fasting insulin, HDL, SBP, DBP

Triacylglycerol: sex, age, age squared, age cubed, WHR, fasting insulin, HDL, LDL, SBP, history of smoking

HDL: sex, age, age squared, age cubed, BMI, BMI squared, WHR, fasting insulin, triacylglycerol, history of diabetes

LDL: sex, age, age squared, age cubed, BMI, BMI squared, WHR, fasting insulin, HDL, triacylglycerol, history of diabetes

SBP: sex, age, age squared, age cubed, BMI, WHR, fasting glucose, DBP, smoking status

DBP: sex, age, age squared, age cubed, BMI, BMI squared, BMI cubed, SBP, smoking status, history of diabetes

^*^ *p*<0.05

**ESM Table 3 – Results of cross-sectional association analyses of binary phenotypes.a**

| Phenotype^b^ | Gene | SNP | Risk allele  (frequency) | Per risk allele  odds ratio (95% CI) | *p* |
| --- | --- | --- | --- | --- | --- |
| History of diabetes | *KCNJ11* | rs5219 | T (0.363) | 0.96 (0.77, 1.21) | 0.753 |
|  | *PPARG* | rs1801282 | C (0.883) | 1.46 (1.00, 2.12) | 0.0496* |
|  | *TCF7L2* | rs7903146 | T (0.303) | 1.27 (1.01, 1.60) | 0.039* |
|  | *IGF2BP2* | rs4402960 | T (0.304) | 1.30 (1.04, 1.63) | 0.022* |
|  | *CDKAL1* | rs10946398 | C (0.303) | 1.03 (0.81, 1.30) | 0.804 |
|  | *SLC30A8* | rs13266634 | C (0.699) | 1.17 (0.92, 1.49) | 0.204 |
|  | *HHEX* | rs1111875 | G (0.584) | 0.98 (0.79, 1.22) | 0.851 |
|  | *FTO* | rs9939609 | A (0.409) | 1.13 (0.91, 1.41) | 0.257 |
| Obesity | *FTO* | rs9939609 | A (0.409) | - | 0.457 |

^a^ Analyses of participants’ history of diabetes were conducted on data from unrelated participants aged 18-80 years (*n*=2,864) at the 1994/95 survey. The analysis of obesity was conducted on data from the whole study population (*n*=4,554) at the 1994/95 survey.

^b^ Phenotype adjustments, full covariate model:

History of diabetes: sex, age, sex × age interaction, age squared, BMI, HDL, LDL, triacylglycerol, SBP, DBP

Obesity: sex, age, age squared, fasting insulin, HDL, LDL, triacylglycerol, SBP, DBP

^*^*p*<0.05

**ESM Table 4 – Results of longitudinal association analyses of lipid levels and BMI.^a^**

|  |  |  |  | Age × SNP interaction | |  | Time × SNP interaction | |
| --- | --- | --- | --- | --- | --- | --- | --- | --- |
| Gene | SNP | Risk allele (frequency) | Factor | Beta (95% CI) | *p* |  | Beta (95% CI) | *p* |
| **Triacylglycerol^b^**  **(log_e_[mmol/l])** | |  |  |  |  |  |  |  |
| *KCNJ11* | rs5219 | T (0.355) | CC | Baseline |  |  |  |  |
|  |  |  | CT | 1.02×10^-3^ (-1.27×10^-3^, 3.31×10^-3^) | 0.38 |  | 1.20×10^-3^ (-1.85×10^-3^, 4.24×10^-3^) | 0.44 |
|  |  |  | TT | 9.94×10^-4^ (-2.36×10^-3^, 4.35×10^-3^) | 0.56 |  | -8.98×10^-4^ (-5.39×10^-3^, 3.59×10^-3^) | 0.70 |
| *PPARG* | rs1801282 | C (0.881) | CC | Baseline |  |  |  |  |
|  |  |  | CG | -1.63×10^-3^ (-4.21×10^-3^, 9.62×10^-4^) | 0.22 |  | 1.02×10^-3^ (-2.45×10^-3^, 4.50×10^-3^) | 0.56 |
|  |  |  | GG | 6.43×10^-3^ (-2.31×10^-3^, 1.52×10^-2^) | 0.15 |  | -1.67×10^-3^ (-1.36×10^-2^, 1.03×10^-2^) | 0.78 |
| *TCF7L2* | rs7903146 | T (0.300) | CC | Baseline |  |  |  |  |
|  |  |  | CT | 1.91×10^-3^ (-3.28×10^-4^, 4.14×10^-3^) | 0.09 |  | 3.52×10^-4^ (-2.62×10^-3^, 3.33×10^-3^) | 0.82 |
|  |  |  | TT | -2.18×10^-3^ (-5.98×10^-3^, 1.63×10^-3^) | 0.26 |  | 5.69×10^-3^ ( 5.53×10^-4^, 1.08×10^-2^) | 0.03^*^ |
| *IGF2BP2* | rs4402960 | T (0.314) | GG | Baseline |  |  |  |  |
|  |  |  | GT | 2.45×10^-3^ ( 1.97×10^-4^, 4.71×10^-3^) | 0.03^*^ |  | -1.76×10^-4^ (-3.17×10^-3^, 2.82×10^-3^) | 0.91 |
|  |  |  | TT | 1.38×10^-3^ (-2.29×10^-3^, 5.06×10^-3^) | 0.46 |  | 6.01×10^-4^ (-4.29×10^-3^, 5.49×10^-3^) | 0.81 |
| *CDKAL1* | rs10946398 | C (0.308) | AA | Baseline |  |  |  |  |
|  |  |  | AC | 1.41×10^-3^ (-8.48×10^-4^, 3.66×10^-3^) | 0.22 |  | 1.43×10^-4^ (-2.86×10^-3^, 3.15×10^-3^) | 0.93 |
|  |  |  | CC | -2.31×10^-3^ (-5.98×10^-3^, 1.36×10^-3^) | 0.22 |  | 3.30×10^-3^ (-1.59×10^-3^, 8.18×10^-3^) | 0.19 |
| *SLC30A8* | rs13266634 | C (0.696) | CC | Baseline |  |  |  |  |
|  |  |  | CT | 4.00×10^-3^ ( 1.76×10^-3^, 6.24×10^-3^) | 0.0005^*^ |  | -2.58×10^-3^ (-5.56×10^-3^, 4.03×10^-4^) | 0.09 |
|  |  |  | TT | 2.71×10^-3^ (-1.10×10^-3^, 6.51×10^-3^) | 0.16 |  | -4.04×10^-3^ (-9.13×10^-3^, 1.05×10^-3^) | 0.12 |
| *HHEX* | rs1111875 | G (0.578) | GG | Baseline |  |  |  |  |
|  |  |  | GA | -1.38×10^-3^ (-3.78×10^-3^, 1.02×10^-3^) | 0.26 |  | -1.31×10^-3^ (-4.50×10^-3^, 1.88×10^-3^) | 0.42 |
|  |  |  | AA | -2.30×10^-3^ (-5.40×10^-3^, 7.94×10^-4^) | 0.15 |  | 2.06×10^-4^ (-3.89×10^-3^, 4.31×10^-3^) | 0.92 |
|  |  |  |  |  |  |  |  |  |
|  |  |  |  |  |  |  |  |  |
| *FTO* | rs9939609 | A (0.402) | TT | Baseline |  |  |  |  |
|  |  |  | TA | -1.61×10^-3^ (-3.96×10^-3^, 7.31×10^-4^) | 0.18 |  | 2.74×10^-3^ (-3.97×10^-4^, 5.87×10^-3^) | 0.09 |
|  |  |  | AA | -5.05×10^-4^ (-3.76×10^-3^, 2.75×10^-3^) | 0.76 |  | 3.33×10^-4^ (-3.98×10^-3^, 4.64×10^-3^) | 0.88 |
| **HDL^b^**  **(log_e_[mmol/l])** |  |  |  |  |  |  |  |  |
| *KCNJ11* | rs5219 | T (0.355) | CC | Baseline |  |  |  |  |
|  |  |  | CT | -4.29×10^-4^ (-1.62×10^-3^, 7.63×10^-4^) | 0.48 |  | 1.62×10^-5^ (-1.59×10^-3^, 1.62×10^-3^) | 0.98 |
|  |  |  | TT | 1.37×10^-4^ (-1.63×10^-3^, 1.91×10^-3^) | 0.88 |  | 2.04×10^-4^ (-2.17×10^-3^, 2.58×10^-3^) | 0.87 |
| *PPARG* | rs1801282 | C (0.881) | CC | Baseline |  |  |  |  |
|  |  |  | CG | 1.80×10^-3^ ( 4.44×10^-4^, 3.15×10^-3^) | 0.01^*^ |  | -2.09×10^-3^ (-3.93×10^-3^, -2.57×10^-4^) | 0.03^*^ |
|  |  |  | GG | -3.83×10^-3^ (-8.24×10^-3^, 5.73×10^-4^) | 0.09 |  | 2.84×10^-3^ (-3.18×10^-3^, 8.86×10^-3^) | 0.36 |
| *TCF7L2* | rs7903146 | T (0.300) | CC | Baseline |  |  |  |  |
|  |  |  | CT | -2.97×10^-4^ (-1.47×10^-3^, 8.72×10^-4^) | 0.62 |  | 3.43×10^-4^ (-1.23×10^-3^, 1.92×10^-3^) | 0.67 |
|  |  |  | TT | 1.49×10^-5^ (-1.96×10^-3^, 1.99×10^-3^) | 0.99 |  | -8.06×10^-4^ (-3.49×10^-3^, 1.87×10^-3^) | 0.56 |
| *IGF2BP2* | rs4402960 | T (0.314) | GG | Baseline |  |  |  |  |
|  |  |  | GT | -5.69×10^-4^ (-1.75×10^-3^, 6.10×10^-4^) | 0.34 |  | 6.82×10^-4^ (-9.00×10^-4^, 2.26×10^-3^) | 0.40 |
|  |  |  | TT | -1.06×10^-3^ (-2.98×10^-3^, 8.61×10^-4^) | 0.28 |  | -1.15×10^-3^ (-3.77×10^-3^, 1.46×10^-3^) | 0.39 |
| *CDKAL1* | rs10946398 | C (0.308) | AA | Baseline |  |  |  |  |
|  |  |  | AC | -4.92×10^-4^ (-1.67×10^-3^, 6.86×10^-4^) | 0.41 |  | 6.75×10^-4^ (-9.12×10^-4^, 2.26×10^-3^) | 0.40 |
|  |  |  | CC | -8.89×10^-4^ (-2.80×10^-3^, 1.02×10^-3^) | 0.36 |  | 2.24×10^-4^ (-2.34×10^-3^, 2.79×10^-3^) | 0.86 |
| *SLC30A8* | rs13266634 | C (0.696) | CC | Baseline |  |  |  |  |
|  |  |  | CT | -8.81×10^-4^ (-2.05×10^-3^, 2.85×10^-4^) | 0.14 |  | 1.32×10^-3^ (-2.53×10^-4^, 2.89×10^-3^) | 0.10 |
|  |  |  | TT | -2.48×10^-3^ (-4.46×10^-3^, -4.89×10^-4^) | 0.01^*^ |  | 2.99×10^-3^ ( 3.21×10^-4^, 5.67×10^-3^) | 0.03^*^ |
| *HHEX* | rs1111875 | G (0.578) | GG | Baseline |  |  |  |  |
|  |  |  | GA | -1.20×10^-3^ (-2.45×10^-3^, 5.15×10^-5^) | 0.06 |  | 1.24×10^-3^ (-4.36×10^-4^, 2.92×10^-3^) | 0.15 |
|  |  |  | AA | -1.38×10^-4^ (-1.75×10^-3^, 1.48×10^-3^) | 0.87 |  | -2.01×10^-4^ (-2.38×10^-3^, 1.98×10^-3^) | 0.86 |
| *FTO* | rs9939609 | A (0.402) | TT | Baseline |  |  |  |  |
|  |  |  | TA | 9.60×10^-4^ (-2.68×10^-4^, 2.19×10^-3^) | 0.13 |  | -8.86×10^-4^ (-2.54×10^-3^, 7.69×10^-4^) | 0.29 |
|  |  |  | AA | 7.17×10^-4^ (-9.75×10^-4^, 2.41×10^-3^) | 0.41 |  | -4.31×10^-4^ (-2.70×10^-3^, 1.84×10^-3^) | 0.71 |
| **BMI^b^ (log_e_[kg/m^2^])** |  |  |  |  |  |  |  |  |
| *KCNJ11* | rs5219 | T (0.355) | CC | Baseline |  |  |  |  |
|  |  |  | CT | 1.85×10^-4^ (-5.21×10^-4^, 8.92×10^-4^) | 0.61 |  | -1.10×10^-4^ (-8.74×10^-4^, 6.54×10^-4^) | 0.78 |
|  |  |  | TT | -3.80×10^-4^ (-1.42×10^-3^, 6.64×10^-4^) | 0.48 |  | -4.40×10^-4^ (-1.57×10^-3^, 6.93×10^-4^) | 0.45 |
| *PPARG* | rs1801282 | C (0.881) | CC | Baseline |  |  |  |  |
|  |  |  | CG | -1.91×10^-4^ (-9.97×10^-4^, 6.16×10^-4^) | 0.64 |  | 2.23×10^-4^ (-6.53×10^-4^, 1.10×10^-3^) | 0.62 |
|  |  |  | GG | 2.50×10^-3^ (-1.78×10^-4^, 5.19×10^-3^) | 0.07 |  | -1.43×10^-3^ (-4.37×10^-3^, 1.51×10^-3^) | 0.34 |
| *TCF7L2* | rs7903146 | T (0.300) | CC | Baseline |  |  |  |  |
|  |  |  | CT | -9.12×10^-5^ (-7.85×10^-4^, 6.03×10^-4^) | 0.80 |  | -2.20×10^-4^ (-9.72×10^-4^, 5.31×10^-4^) | 0.57 |
|  |  |  | TT | -6.18×10^-4^ (-1.79×10^-3^, 5.57×10^-4^) | 0.30 |  | 1.51×10^-4^ (-1.12×10^-3^, 1.42×10^-3^) | 0.82 |
| *IGF2BP2* | rs4402960 | T (0.314) | GG | Baseline |  |  |  |  |
|  |  |  | GT | -3.86×10^-4^ (-1.09×10^-3^, 3.13×10^-4^) | 0.28 |  | 4.92×10^-4^ (-2.65×10^-4^, 1.25×10^-3^) | 0.20 |
|  |  |  | TT | -9.53×10^-5^ (-1.23×10^-3^, 1.04×10^-3^) | 0.87 |  | 2.06×10^-4^ (-1.02×10^-3^, 1.43×10^-3^) | 0.74 |
| *CDKAL1* | rs10946398 | C (0.308) | AA | Baseline |  |  |  |  |
|  |  |  | AC | -3.29×10^-4^ (-1.03×10^-3^, 3.69×10^-4^) | 0.36 |  | 2.22×10^-4^ (-5.33×10^-4^, 9.77×10^-4^) | 0.56 |
|  |  |  | CC | -8.82×10^-5^ (-1.23×10^-3^, 1.06×10^-3^) | 0.88 |  | -7.27×10^-4^ (-1.97×10^-3^, 5.15×10^-4^) | 0.25 |
| *SLC30A8* | rs13266634 | C (0.696) | CC | Baseline |  |  |  |  |
|  |  |  | CT | -8.79×10^-5^ (-7.81×10^-4^, 6.06×10^-4^) | 0.80 |  | -6.23×10^-5^ (-8.13×10^-4^, 6.88×10^-4^) | 0.87 |
|  |  |  | TT | 1.05×10^-3^ (-1.29×10^-4^, 2.22×10^-3^) | 0.08 |  | -6.95×10^-4^ (-1.97×10^-3^, 5.84×10^-4^) | 0.29 |
| *HHEX* | rs1111875 | G (0.578) | GG | Baseline |  |  |  |  |
|  |  |  | GA | 2.71×10^-4^ (-4.74×10^-4^, 1.02×10^-3^) | 0.48 |  | -1.38×10^-4^ (-9.42×10^-4^, 6.67×10^-4^) | 0.74 |
|  |  |  | AA | 1.28×10^-3^ ( 3.28×10^-4^, 2.23×10^-3^) | 0.01^*^ |  | -1.07×10^-3^ (-2.09×10^-3^, -4.14×10^-5^) | 0.04^*^ |
| *FTO* | rs9939609 | A (0.402) | TT | Baseline |  |  |  |  |
|  |  |  | TA | 2.58×10^-4^ (-4.70×10^-4^, 9.86×10^-4^) | 0.49 |  | 2.06×10^-5^ (-7.68×10^-4^, 8.10×10^-4^) | 0.96 |
|  |  |  | AA | -2.05×10^-5^ (-1.03×10^-3^, 9.88×10^-4^) | 0.97 |  | -2.79×10^-4^ (-1.37×10^-3^, 8.09×10^-4^) | 0.61 |

^a^ Analyses were conducted on data from unrelated participants aged 18-80 years (*n*=2,864). Results are for age × SNP and time × SNP interaction terms with co-dominant genetic models.

^b^ Phenotype adjustments:

Triacylglycerol: sex, age, age × sex, BMI, smoking status, age at first survey, time of survey

HDL: sex, age, age × sex, BMI, smoking status, age at first survey, time of survey

BMI: sex, age, age × sex, smoking status, age at first survey, time of survey

^*^ *p*<0.05
